# Supplementary material for: Blockade of Platelet CysLT1R Receptor with Zafirlukast Counteracts Platelet Protumoral Action and Prevents Breast Cancer Metastasis to Bone and Lung
Source: Int J Mol Sci. 2022 Oct 13;23(20):12221. doi: 10.3390/ijms232012221 (PMC9603002; doi:10.3390/ijms232012221)
Supplement: Supplementary file 1 [file ijms-23-12221-s001.zip › ijms-1906112-supplementary.pdf]

Supplementary Figures

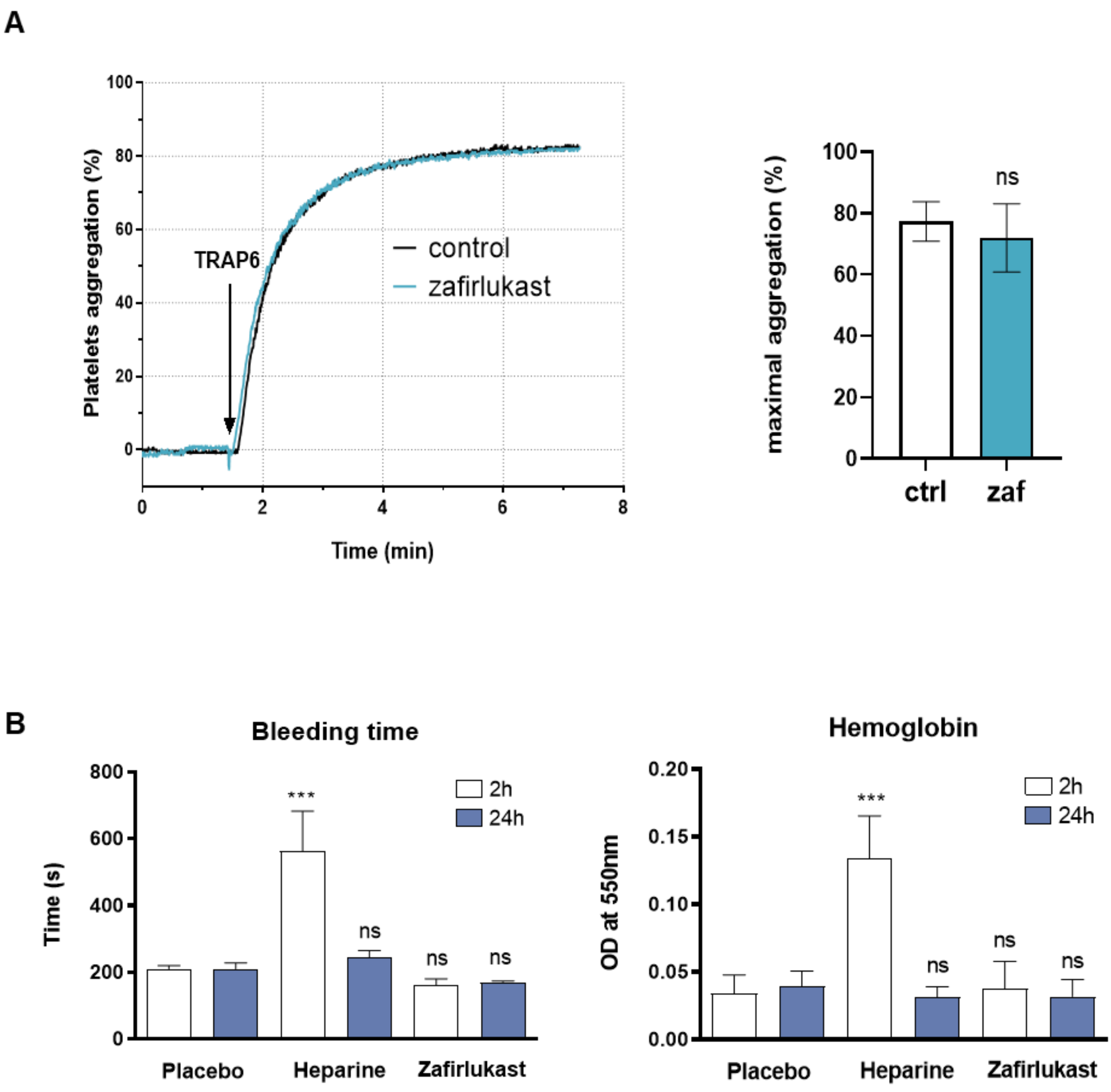

Figure S1. CyLT1R blockade with zafirlukast does not affect platelet physiological functions.
A. CyLT1R blockade with zafirlukast does not affect TRAP6-induced platelets aggregation. Human platelets aggregation was induced by the addition of 15µM of the platelet agonist TRAP6 and was measured by light transmission aggregometry. The prior addition of 10µM zafirlukast does not affect the latency time or the maximal aggregation induced by TRAP6. **B.** CyLT1R blockade with zafirlukast does not affect hemostasis in mice. Mice received orally physiological serum (placebo), the anticoagulant heparine (50IU/kg), or the CysLT1R antagonist zafirlukast (0,4mg/kg) then their tail was incised 2h or 24h after the treatment to measure bleeding time and hemoglobine release. *** $p < 0.001$.

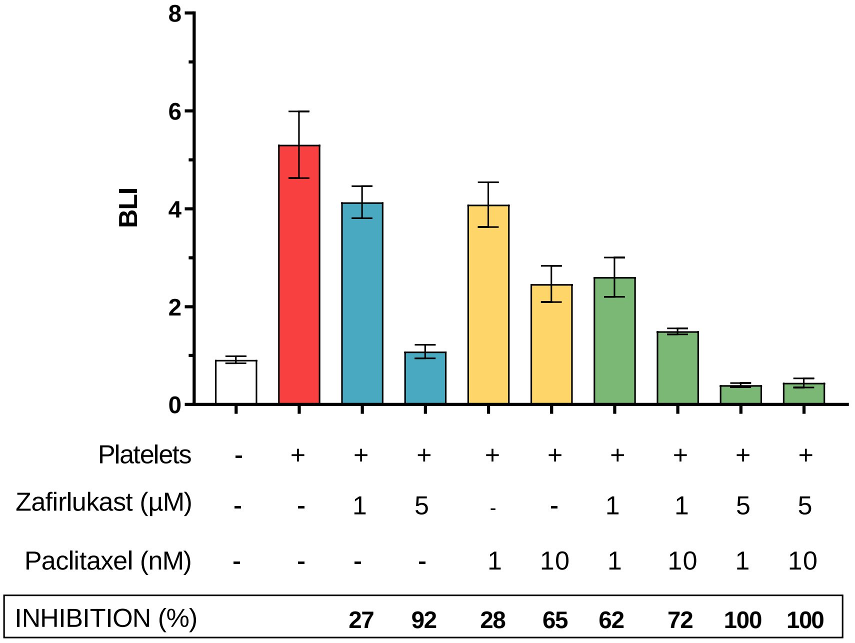

Figure S2. Zafirlukast and paclitaxel show additive chemopreventive effects *in vitro* on MDA-B02-Luc cancer cells survival.

MDA-B02-Luc breast cancer cells were cultured for 48h with human platelets in presence of zafirlukast and/or paclitaxel. The survival of cancer cells is measured using a bioluminescence assay (BLI).

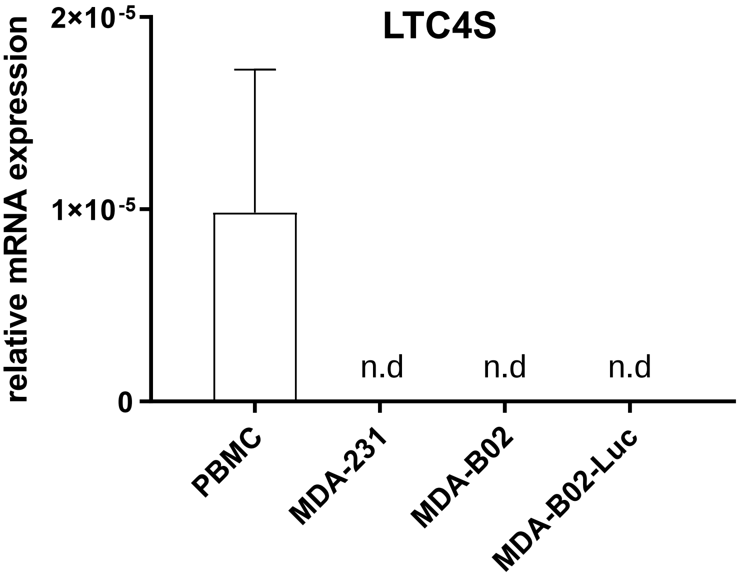

Figure S3. LTC4S expression is not detected by RT-qPCR in our cancer cell lines. RT-qPCR analysis of LTC4S mRNA. Data is the mean ±SD (n=3). Peripheral blood mononuclear cells (PBMC) were used as a positive control of LTC4S expression. N.d= not detected.

Supplementary Materials and Methods

Platelet aggregation

Washed platelets aggregation was measured by light aggregometry. In brief, platelet samples ($3x10^8$/mL) were placed in a lumi-aggregometer (Chronolog) and incubated for 1 min at 37°C, with stirring at 1000rpm, prior to the addition of aggregation agent, TRAP6 at 15µM. To study the effect of CysLT1R blockade on platelet aggregation, zafirlukast at 10µM was added 1min prior the addition of the aggregation agent. The reaction was monitored and analyzed by the Aggro-Link software (Chronolog).

Tail-bleeding and hemoglobin assay in mice

The effect of zafirlukast on hemostasis was evaluated *in vivo* in mice. Mice received oraly physiological serum (placebo), the anticoagulant heparine (50IU/kg) or zafirlukast (0.4mg/kg) then a 10mm segment of the tail was amputated with a scalpel 2h or 24h after treatment. The tail was immediately immersed in a 50mL Falcon tube containing isotonic saline pre-warmed to 37°C to measure the time when bleeding ceased. The hemoglobin release in the saline solution was measured spectrophotometrically at 550nm.
